# Heritability and Clinical Determinants of Serum Indoxyl Sulfate and *p*-Cresyl Sulfate, Candidate Biomarkers of the Human Microbiome Enterotype

**DOI:** 10.1371/journal.pone.0079682

**Published:** 2014-05-21

**Authors:** Liesbeth Viaene, Lutgarde Thijs, Yu Jin, Yanping Liu, Yumei Gu, Björn Meijers, Kathleen Claes, Jan Staessen, Pieter Evenepoel

**Affiliations:** 1 Department of Medicine, Division of Nephrology, Dialysis and Renal Transplantation, University Hospital Leuven, Leuven, Belgium; 2 Studies Coordinating Centre, Division of Hypertension and Cardiovascular Rehabilitation, Department of Cardiovascular Diseases, University of Leuven, Leuven, Belgium; 3 Department of Epidemiology, Maastricht University, Maastricht, Netherlands; IRCSS - Istituto di Ricerche Farmacologiche Mario Negri, Italy

## Abstract

**Background:**

Indoxyl sulfate and *p*-cresyl sulfate are unique microbial co-metabolites. Both co-metabolites have been involved in the pathogenesis of accelerated cardiovascular disease and renal disease progression. Available evidence suggests that indoxyl sulfate and *p*-cresyl sulfate may be considered candidate biomarkers of the human enterotype and may help to explain the link between diet and cardiovascular disease burden.

**Objective and Design:**

Information on clinical determinants and heritability of indoxyl sulfate and *p*-cresyl sulfate serum is non-existing. To clarify this issue, the authors determined serum levels of indoxyl sulfate and *p*-cresyl sulfate in 773 individuals, recruited in the frame of the Flemish Study on Environment, Genes and Health Outcomes (FLEMENGHO study).

**Results:**

Serum levels of indoxyl sulfate and *p*-cresyl sulfate amounted to 3.1 (2.4–4.3) and 13.0 (7.4–21.5) μM, respectively. Regression analysis identified renal function, age and sex as independent determinants of both co-metabolites. Both serum indoxyl sulfate (h^2^ = 0.17) and *p*-cresyl sulfate (h^2^ = 0.18) concentrations showed moderate but significant heritability after adjustment for covariables, with significant genetic and environmental correlations for both co-metabolites.

**Limitations:**

Family studies cannot provide conclusive evidence for a genetic contribution, as confounding by shared environmental effects can never be excluded.

**Conclusions:**

The heritability of indoxyl sulfate and *p*-cresyl sulfate is moderate. Besides genetic host factors and environmental factors, also renal function, sex and age influence the serum levels of these co-metabolites.

## Introduction

The human intestinal tract is colonized by hundreds of trillions of microbes, which collectively possess hundreds of times as many genes as coded for by the human genome. The combined genetic potential of the endogenous flora is referred to as the ‘microbiome’ [1]. The dissimilarity in gut bacterial composition between individuals is huge [2]. Recent findings demonstrate that the variation in the microbiome of individuals is not continuous, but stratified [3], indicating that one's individual gut flora are not a randomly composed set of bacteria but one of several possible well-balanced ecosystems. The microbiome can be classified into just three broad “enterotypes” dominated by three different genera: *Bacteroides*, *Prevotella* and *Ruminococcus* [3].

It is increasingly recognized that the microbiome may affect health and disease of the host, e.g. by modulating the immune system, by harvesting energy from the breakdown of otherwise difficult to digest plant glycans, by synthesizing vitamins, by metabolizing xenobiotics or by exposing the host to potentially toxic metabolites [4]. Metabolites derived from the microbial community are referred to as co-metabolites. This metabolic phenotype provides a readout of the metabolic state of an individual and is the product of genetic and environmental (diet, liefestyle, gut microbial activity) contributions under a particular set of conditions [5].

Indoxyl sulfate and *p*-cresyl sulfate are the sulfate conjugates of indole and *p*-cresol, which are end-products of bacterial protein fermentation of respectively tryptophan and tyrosine in the colon [6], [7]. Targeted and untargeted metabolomics-based investigations in mice and humans identified indoxyl sulfate and *p*-cresyl sulfate as unique microbial co-metabolites [8], [9] and emphasized the major impact of diet on their generation [10], [11]. Indeed, p-cresol and indole were identified as co-metabolites showing the most pronounced increase in healthy volunteers exposed to a high-protein diet [10], [12]. It should be of note, the bulk of indole and *p-*cresol produced in the colon is absorbed, with less than 5% excreted in faeces [13].

In vitro and ex vivo data show that indoxyl sulfate and *p*-cresyl sulfate may trigger or accelerate cardiovascular disease and progression of kidney failure [14]–[19]. Clinical observational studies also relate high levels of both metabolites with overall mortality [19]–[22] as well as cardiovascular disease [23], [24] and renal disease progression [25].

Altogether, these data support the hypothesis that indoxyl sulfate and *p*-cresyl sulfate may be considered candidate biomarkers of the human microbiome enterotype and may help to explain the link between diet and cardiovascular disease burden

To our knowledge, information on the clinical determinants and heritability of indoxyl sulfate and *p*-cresyl sulfate serum concentrations does not exist in an unbiased randomly recruited population study. The current study addresses these issues.

## Subjects and Methods

### Study population

From August 1985 until November 1990, a random sample of the households living in a geographically defined area of Northern Belgium was invited, with the goal of recruiting equal numbers of participants in each of six subgroups defined by sex and age (20–39, 40–59, and ≥60 years) that would go through repeated examination cycles. The study population included 2,310 subjects. The participation rate among the subjects contacted was 66.1%. The random subsample for the present analysis included 818 subjects invited for a follow-up examination. Our study sample included 112 unrelated subjects and 661 subjects from 80 complex pedigrees with a median size of 5 individuals (range: 2 to 38) and encompassing 1 (n = 18 pedigrees), 2 (n = 45) or 3 (n = 17) generations. In 44 of these participants we did not have serum available for analysis. In addition, indoxyl sulfate and *p*-cresyl sulfate were below the detection limit in 1 and 23 subjects respectively. The analyses therefore included 773 subjects for indoxyl sulfate and 751 subjects for *p*-cresyl sulfate. The Ethics Committee of the University of Leuven approved the protocol of FLEMENGHO (Flemish Study on Environment, Genes and Health Outcomes). All subjects gave informed written consent.

### Measurements

Subjects were fasted for at least 6 hours. Trained nurses measured blood pressure and anthropometric characteristics. They administered a questionnaire to collect information about each subject's medical history, smoking and drinking habits, and intake of medications. Each participant's office blood pressure was the average of five consecutive readings. Elevated blood pressure was a systolic blood pressure above 140 mmHg and/or 90 mmHg diastolic or use of antihypertensive drug treatment. Body mass index (BMI) was weight in kilograms divided by the square of height in metres. Myocardial infarctions, stroke, transient ischemic attack, coronary bypass surgery, percutaneous angioplasty and peripheral arterial disease were enclosed in the evaluation of cardiovascular complications. Blood glucose, total, HDL and LDL cholesterol, triglycerides, and serum creatinine were also measured in all subjects by routine laboratory methods. Glomerular filtration rate was estimated using the Cockcroft-Gault formula and the creatinine clearance was calculated form 24 hour urinary collections. Serum total indoxyl sulfate and *p*-cresyl sulfate were measured using high-performance liquid chromatography, as previously described [26]. The intra-and inter-assay variability were all below 5%. The limit of quantification for indoxyl sulfate and p-cresyl sulfate were respectively 2.39 and 7.36 µM. The day-to-day variability of toxins levels was tested in a separate cohort of 10 healthy individuals (6 males, age 32.8 year) and was 25% for *p*-cresyl sulfate and 27% for indoxyl sulfate.

Unfortunately, individual dietary data are missing in the present cohort. According to a recent national nutritional survey, the mean total energy intake in Belgium for adults between 19–59 years is 2578 and 1680 kcal/day for respectively male and female individuals. Protein, carbohydrate and fat intake is as follows: 16%, 38% and 46%. Data on dietary fiber intake in this survey are lacking, but fruit and vegetable intake, overall, is considered too low as in most Western countries [27].

### Statistical methods

Statistical analyses were performed using SAS software version 9.2 (SAS Institute, Cary, NC, USA). A p-value of less than 0.05 was considered to be statistically significant. Continuous data are presented as mean ± standard deviation and categorical data as frequencies and percentages. Comparison between subjects according to quartiles of indoxyl sulfate or *p*-cresyl sulfate concentrations was performed by the large sample Z-test for continuous variables and the χ^2^-test for categorical variables. For determining upper limits of both toxins, all participants (in specified age groups) with a measured creatinine clearance >90 ml/min were considered for analysis and the 95th percentile was calculated. We searched for possible determinants of indoxyl sulfate or *p*-cresyl sulfate concentrations by a stepwise regression procedure with p-values for independent variables to enter and to stay in the models set at 0.15. To describe determinants of indoxyl sulfate and *p*-cresyl sulfate, a stepwise regression model not taking into account family relationship was used to select covariables. Then, a mixed model with family included as a random effect and the covariables selected in the previous step entered as fixed effects was used to calculate the parameter estimates. To estimate heritability and to calculate the genetic and environmental correlations, we used Statistical Analysis for Genetic Epidemiology software (S.A.G.E. 2009) package. The maximum likelihood method as implemented in the ASSOC procedure of S.A.G.E. was applied. We estimated heritability by assuming multivariate normality after a simultaneously estimated power transformation. ASSOC uses a multiple linear regression model, in which the residual variance is partitioned into the sum of an additive polygenic component, a sibling component, a marital effect and an individual-specific random component. Heritability (h^2^) was estimated as the polygenic component divided by the total residual variance. The proportion of the variance explained by shared environmental effects was estimated as the marital component divided by the total variance.

## Results

### Characteristics of the participants

**Figure 1:**
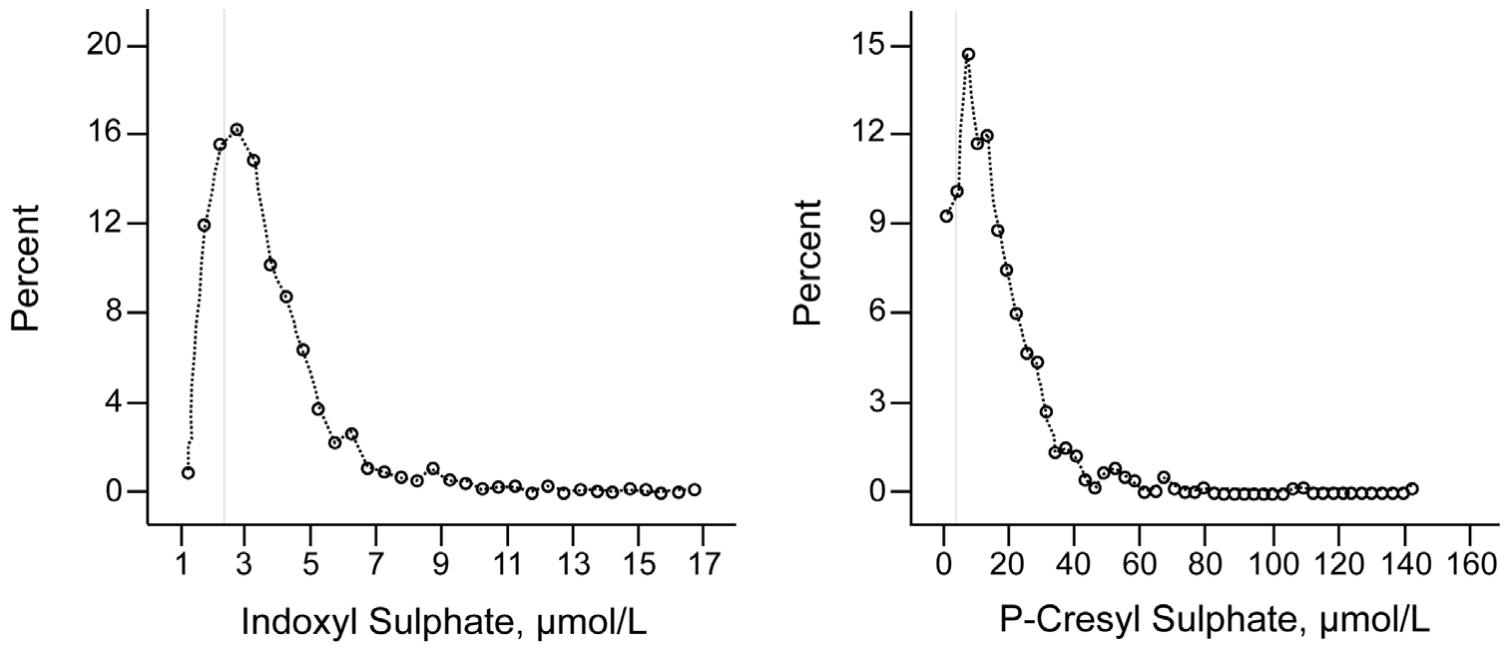
Distribution of indoxyl sulfate and *p*-cresyl sulfate. The vertical line is the limit of quantification.

**Figure 2:**
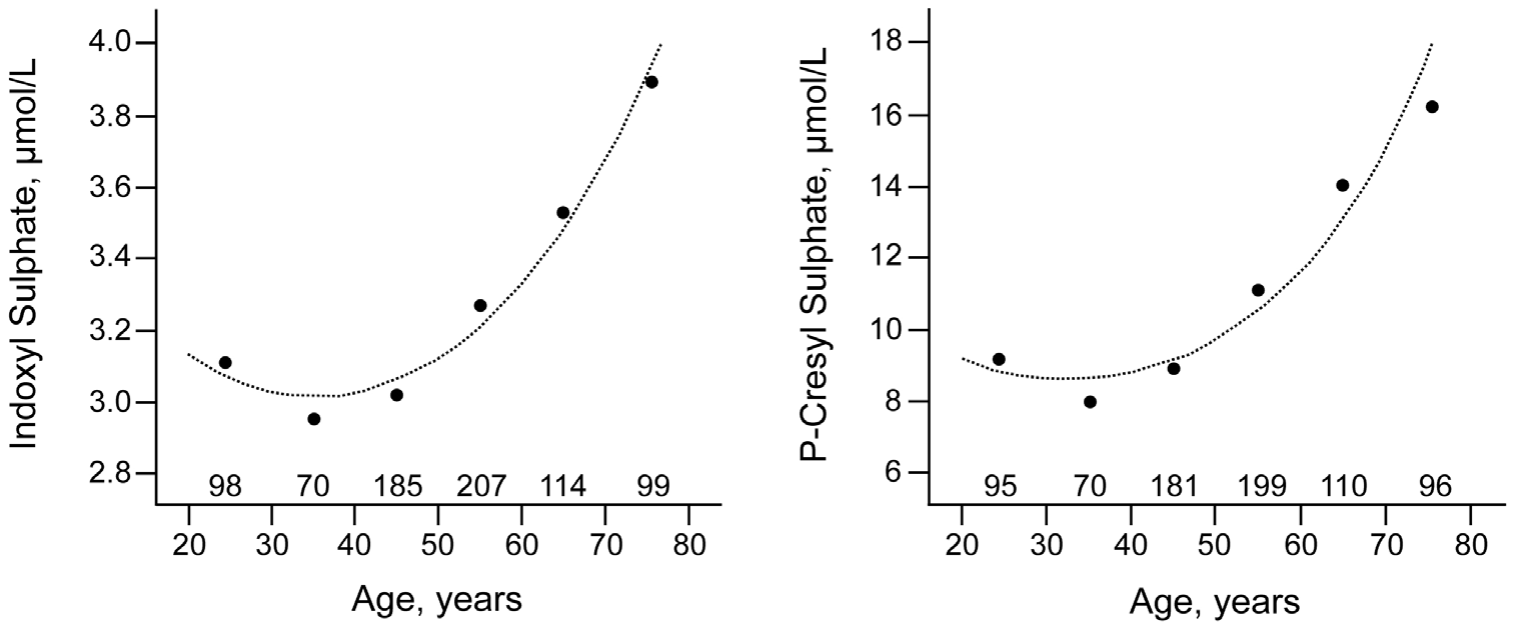
Indoxyl sulfate and *p*-cresyl sulfate according to age. The dots indicate the geometric means of indoxyl sulfate (IndS) and p-cresyl sulfate (PCS) in decades of age (<30 years, 30–39 years, 40–49 years, 50–59 years, 60–69 years and ≥70 years). The numbers above the horizontal axis are the number of subjects in the various age classes. The curves are calculated from a regression model with log IndS and log PCS as dependent variables and age and age-squared as independent variables. For IndS the P-values of the linear and squared terms were 0.035 and 0.0024 respectively. The corresponding P-values for PCS were 0.070 and 0.004.

Characteristics of the participants are summarized in Table 1 and 2. Half of them were men. Mean age was 51.1 years. 20% of the study population smoked. Body mass index was 26.5 kg/m^2^. Mean blood pressure was within the normal range. The median serum concentration amounted to 3.19 µM (iqr 2.39–4.27) for indoxyl sulfate and to 13.03 µM (iqr 7.36–21.48) for *p*-cresyl sulfate. Distribution range for serum concentration of either toxin was skewed with respectively 26% (indoxyl sulfate) and 10% (*p*-cresyl sulfate) of the participants being at the limit of quantification (LOQ) (Figure 1). After logarithmic transformation, a distribution approximating a normal one was achieved for both variables. Serum concentrations of indoxyl sulfate and *p-*cresyl sulfate significantly increased with age. Figure 2 describes toxin concentrations by decades of age, revealing a highly significant quadratic association between either toxin concentration and age. A significant increase in indoxyl sulfate and *p-*cresyl sulfate serum concentrations was observed starting from the age of 50. The 95^th^ percentiles for participants below 50 years were 5.9 and 31.9 µM for indoxyl sulfate and *p*-cresyl sulfate respectively; between 50 and 60 years, these percentiles were 7.8 and 36.9 µM and above 60 years 7.1 and 49.0 µM. Serum concentrations of indoxyl sulfate and *p*-cresyl sulfate correlated significantly (r = 0.48; p<0.0001).

**Table 1:** Baseline characteristics by quartiles of indoxyl sulfate.

	Indoxyl Sulfate, µmol/L				
Characteristics	<2.39 (n = 197)	2.39–3.149 (n = 192)	3.150–4.275 (n = 192)	>4.275 (n = 192)	*P*
Characteristic n (%)					
Men	100 (50.8)	86 (44.8)	88 (45.8)	110 (57.3)	0.206
Hypertension	67 (33.8)	77 (40.1)	77 (40.1)	97 (50.1)	0.01
Antihypertensive drug intake	33 (16.8)	43 (22.4)	51 (26.6)	68 (35.4)	<0.0001
Diabetes mellitus	4 (2.0)	6 (3.1)	5 (2.6)	12 (6.3)	0.044
Current smokers	55 (27.9)	44 (22.9)	32 (16.7)	26 (13.5)	0.0002
Current drinkers	156 (79.2)	127 (66.1)	118 (61.5)	132 (68.8)	0.014
History of CV disease	14 (7.1)	18 (9.4)	16 (8.3)	24 (12.5)	0.106
Age-adjusted characteristics					
Body mass index, kg/m^2^	26.2(0.30)	26.5(0.31)	26.6(0.30)	26.8(0.31)	0.507
Systolic blood pressure, mmHg	129.7(1.04)	127.7(1.04)	128.4(1.04)	132.0(1.05)	0.023
Diastolic blood pressure, mmHg	80.1(0.64)	78.9(0.65)	79.6(0.65)	81.1(0.65)	0.104
Serum cholesterol, mmol/L	5.2(0.066)	5.2(0.066)	5.2(0.066)	5.4(0.067)	0.170
Triglycerides, mmol/L	1.7(0.076)	1.8(076)	1.8(076)	2.2(077)	<0.0001
Serum creatinine, µmol/L	82.4(1.11)	81.6(1.11)	82.8(1.11)	90.3(1.12)	<0.0001
Measured creatinine clearance, ml/sec/1.73m^2^	1.7(0.041)	1.7(0.041)	1.8(0.041)	1.7(0.041)	0.852
Calculated creatinine clearance, ml/min/1.73m^2^	93.6(1.63)	94.9(1.64)	94.5(1.64)	90.9(1.65)	0.304

P-value for trend across the quartiles is given. Values are mean (SD) or number of participants (%) as appropriate.

**Table 2:** Baseline characteristics by quartiles of *p*-cresyl sulfate.

	P-Cresyl Sulfate, µmol/L				
Characteristics	<7.36 (n = 188)	7.36–13.02 (n = 188)	13.03–21.43 (n = 187)	>21.43 (n = 188)	*P*
Characteristics n (%)					
Men	101 (53.7)	91 (48.4)	92 (49.2)	91 (48.4)	0.35
Hypertension	72 (38.6)	61 (32.3)	84 (44.9)	89 (47.3)	0.08
Antihypertensive drug intake	40 (21.3)	30 (16.0)	56 (29.9)	60 (31.9)	0.0012
Diabetes mellitus	6 (3.2)	2 (1.1)	9 (4.8)	7 (3.7)	0.35
Current smokers	44 (23.4)	48 (25.5)	37 (19.8)	23 (12.2)	0.0029
Current drinkers	135 (71.8)	138 (73.4)	135 (72.2)	109 (58.0)	0.0048
History of CV disease	16 (8.5)	17 (9.0)	13 (7.0)	25 (13.3)	0.20
Age-adjusted characteristics					
Body mass index, kg/m^2^	26.6 (0.31)	26.5 (0.31)	26.6 (0.31)	26.3 (0.32)	0.749
Systolic blood pressure, mmHg	131.0 (1.07)	128.4 (1.07)	128.7 (1.06)	129.5 (1.09)	0.317
Diastolic blood pressure, mmHg	80.4 (0.67)	79.7 (0.66)	80.2 (0.66)	79.5 (0.68)	0.775
Serum cholesterol, mmol/L	5.2 (0.068)	5.2 (0.067)	5.3 (0.067)	5.3 (0.069)	0.960
Triglycerides, mmol/L	1.9 (0.079)	1.8 (078)	1.8 (078)	1.9 (080)	0.877
Serum creatinine, µmol/L	82.9 (1.16)	82.7 (1.15)	83.6 (1.15)	88.3 (1.18)	0.0019
Measured creatinine clearance, ml/min/1.73m^2^	1.78 (0.041)	1.71 (0.041)	1.78 (0.041)	1.63 (0.042)	0.041
Calculated creatinine clearance, ml/min/1.73m^2^	96.7 (1.66)	94.4 (1.65)	94.1 (1.65)	88.5 (1.68)	0.0062

The P-value for trend across the quartiles is given. Values are mean±SD or number of participants (%) as appropriate.

### Determinants of serum indoxyl sulfate and *p*-cresyl sulfate concentrations

Current smokers had significantly lower levels of both indoxyl sulfate and *p*-cresyl sulfate, as had current drinkers. Diabetic patients were more likely to have higher indoxyl sulfate concentrations. Higher concentrations of indoxyl sulfate were associated with increased body mass index, systolic blood pressure, serum triglycerides and lower renal function (Table S1 and S2 in File S1). After adjustment for age, only higher serum triglycerides and systolic blood pressure were associated with higher indoxyl sulfate levels (Table 1). Higher *p*-cresyl sulfate concentrations were only associated with lower renal function after age adjustment (Table 2). As shown in Table 3, indoxyl sulfate was 7.2% (95% confidence interval [CI]: 0.1%, 14.8%) higher in men compared to women, 8.4% (CI: 1.5%, 14.7%) lower in smokers than in non-smokers and 7.5% (CI: 1.2%, 13.4%) lower in drinkers than in non-drinkers. In addition, indoxyl sulfate increased with 4.7% (CI: −13.7%, 2 7.2%) from 40 to 60 years, with 16.2% (CI: −3.5%, 39.8%) from 60 to 80 years, with 8.2% (CI: 5.9%, 10.6%) for each 10 µmol/l increase in serum creatinine and with 7.9% (CI: 3.4%, 12.5%) for a doubling in triglycerides. p-Cresyl sulfate on the other hand was 24.3% (CI: 4.4%, 48.0%) higher in men than in women, increased with 36.0% (CI: −15.7%, 119.3%) from 40 to 60 years and with 94.9% (CI: 30.7%, 190.8%) from 60 to 80 years, decreased with 6.4% (CI: 1.3%, 11.3%) per 10 mmHg increase in systolic blood pressure and increased with 12.9% (CI: 6.7%, 19.5%) for each 10 µmol/l increase in serum creatinine.

**Table 3:** Determinants of indoxyl sulfate and *p*-cresyl sulfate.

	Log Indoxyl sulfate	Log *p*-Cresyl sulfate
**N**	773	751
**Variance explained by pedigree**	0.054	0.046
**Variance explained by fixed effects**	0.153	0.069
**Parameter estimates**		
Intercept	0.268±0.0782^***^	0.916±0.247^***^
Male sex	0.0302±0.0152*	0.0945±0.0387*
Age, years	−0.00461±0.00225*	−0.0128±0.00605*
Age squared, years^2^	0.000056±0.000022*	0.000195±0.000061^**^
Current smoking	−0.0379±0.0159*	NS
Current alcohol intake	−0.0337±0.0146*	NS
Conventional systolic blood pressure	NS	−0.00289±0.00119*
Log triglycerides, mmol/L	0.120±0.0310^***^	NS
Serum creatinine, µmol/L	0.00344±0.000475^ 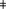 ^	0.0527±0.0126^ 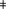 ^

^*^P<0.05; ^**^P<0.01; ^***^P<0.001; ^
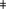
^P<0.0001.

First a stepwise regression model not taking into account family relationships was used to select the covariables. Then, a mixed model with family included as a random effect and the covariables selected in the previous step entered as fixed effects was used to calculate parameter estimates. The following variables were offered to the stepwise regression model: sex, age (linear and squared term), systolic and diastolic blood pressure, history of cardiovascular disease, antihypertensive drug intake, current smoking and alcohol intake, body mass index, diabetes mellitus, serum total cholesterol, triglycerides, serum creatinine and calculated creatinine clearance.

### Heritability analysis

Indoxyl sulfate (h^2^ = 0.16) as well as *p*-cresyl sulfate (h^2^ = 0.24) concentrations showed significant heritability, even after full adjustment for covariables (*p* = 0.02) (Table 4). For comparison, the sex- and age-adjusted heritability was 0.81 for body height and 0.55 for body weight. As shown in Table S3 in File S1, the intra-familial correlation coefficients were significant for *p*-cresyl sulfate and indoxyl sulfate in parent-offspring pairs living together only (Table S3 in File S1). However, it should be of note that the intra-familial correlation coefficients were similar (p>0.05) amongst pairs living together and pairs living apart.

**Table 4:** Heritability of indoxyl sulfate and *p*-cresyl sulfate.

	Unadjusted		Age-adjusted		Fully adjusted 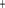	
	h^2^	P	h^2^	P	h^2^	P
Indoxyl sulfate	0.20±0.085	0.011	0.20±0.084	0.010	0.16±0.080	0.023
p-Cresyl sulfate	0.22±0.085	0.005	0.23±0.087	0.004	0.24±0.119	0.024

Values are proportions ± standard error. Indoxyl sulfate and *p*-cresyl sulfate were log-transformed.

h^2^, heritability.

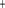
Adjusted for sex, age (linear and squared term), triglycerides, current smoking status, measured creatinine clearance and history of cardiovascular complications.

## Discussion

The present study aimed to evaluate clinical determinants and heritability of indoxyl sulfate and *p*-cresyl sulfate serum concentrations in a randomly selected cohort of white European population.

Serum levels of indoxyl sulfate and *p*-cresyl sulfate reflect the balance between generation and elimination. Indoxyl sulfate and *p*-cresyl sulfate both originate uniquely from colonic microbial metabolism. Indoxyl sulfate and *p*-cresyl sulfate are the sulfate conjugates of indole and *p*-cresol, which are end-products of bacterial protein fermentation of respectively tryptophan and tyrosine in the colon [6], [7]. A “Western” diet, rich in protein and low in dietary fiber, is associated with an increased generation of these co-metabolites [6], [10], [28], whereas a “Mediterranean” diet, characterized by a high consumption of fruit and vegetables, a high consumption of complex carbohydrates, a moderate consumption of fish, and a high consumption of olive oil, suppresses the generation of indoxyl sulfate and *p*-cresyl sulfate [13], [29], [30]. Elimination of these co-metabolites occurs mainly through proximal renal tubular secretion mediated by organic anion transporters (OATs). As changes in the structure or function of the microbiome were shown to contribute to the pathogenesis of various diseases, we aimed to study the determinants of the co-metabolites indoxyl sulfate and *p*-cresyl sulfate, which may accelerate cardiovascular disease and progression of kidney failure, in the general population. Regression analysis identified renal function, age and sex as independent determinants of serum levels of both co-metabolites.

The dependence of the serum concentrations of indoxyl sulfate and *p*-cresyl sulfate on renal function is expected as both co-metabolites are well-known uremic retention molecules [31]. Moreover, recent evidence indicates that uremia per se may profoundly alter the composition of the gut microbiome [32]. In line with the latter, we observed increased generation of *p*-cresyl sulfate along the progression of chronic kidney disease [33].

The association between the serum concentrations of indoxyl sulfate and *p*-cresyl sulfate and age is remarkable and intriguing and confirms previous observations in chronic kidney disease patients [23]. These observations support the hypothesis that aging goes along with a trend towards the *Bacteroides* enterotype and thus more prominent proteolytic fermentation and essentially concur with data from previous “classical” microbiology studies [34], [35]. Whether these changes are related to a reduced immune function or whether they are due to concomitant changes in nutrition, gastrointestinal tract physiology, comorbidity and use of medication with advancing age remains to be established.

Serum concentrations of indoxyl sulfate and *p*-cresyl sulfate were also found to be higher in men. Although this finding may reflect differences in the composition of gut microbiota between men and women, available evidence argues against this explanation [3]. Gender differences in intestinal absorption, metabolism and/or renal clearances could also be involved, but supporting evidence, at least in humans, is lacking so far.

Smoking and alcohol consumption were associated with lower indoxyl sulfate levels and *p*-cresyl sulfate, although for the latter, the association was lost in multivariable analyses. The mechanisms underlying these associations are unclear. Although a direct impact on the microbiome is possible [36], [37], specific behavioral characteristics related to smoking and alcohol consumption may also be involved. However, since smokers consume more energy and fat and less fiber than nonsmokers [38] and since alcohol and smoking may result in the formation of metabolites that might compete with other organic anions for their elimination, a direct instead of an inverse relationship between smoking and metabolites levels would be expected.

Whether the independent and direct association between indoxyl sulfate and serum triglycerides reflects OAT lipotoxicity [39], or alternatively reflects a dietary link requires further investigation.

Serum levels of indoxyl sulfate and *p*-cresyl sulfate significantly correlated. Given the similarities in origin and elimination kinetics, this correlation was not unexpected. The strength of the correlation was however rather weak (r = 0.48) and clinical determinants of both co-metabolites did slightly differ. This suggests that the (bacterial) metabolism of both metabolites though similar is not identical and corroborates findings in other cohorts [25], [40].

Literature data indicate that host genetics may influence the composition of the microbiota, e.g. by influencing the environmental conditions of the habitat, such as length of the intestine and transit time [41]. Gastrointestinal tract physiology is a powerful predictor of the bacterial community composition of feces [1] and colonic transit time significantly and directly correlates with the generation rate of *p*-cresol [28]. Results from studies evaluating microbiota in monozygotic and dizygotic twins however were not concordant [42]. To the best of our knowledge, the present study is the first to implement an adequate powered analysis of the heritability of the presented examined 2 co-metabolites. Heritability analyzes the relative contributions of differences in genetic and non-genetic factors to the phenotypic variance in a population. The ASSOC method uses a multiple linear regression model, in which the residual variance is partitioned into the sum of an additive polygenetic effect, a sibling effect, a marital effect and an individual-specific effect. Heritability, according to this method, is estimated as the polygenic component divided by the total variance. The heritability (h^2^) of the serum indoxyl sulfate and *p*-cresyl sulfate levels in fully adjusted models was 17% and 18% respectively. These estimates of heritability are modest. For comparison, the total phenotypic correlation between body height and weight was 0.41. Secondly, it also needs to be emphasized that heritability is a population and situation-specific parameter. Certain population-specific characteristics may influence estimates of heritability obtained by variance component analysis, despite an identical underlying biologic mechanism across populations. For example, a genetically homogenous population will produce a lower estimate than a genetically heterogeneous population, while a population with a greater diversity of environmental factors will often produce a lower heritability than will one with a more homogeneous environment. The present study sample was recruited from a geographically defined area in northern Belgium. Thus, the genetic heterogeneity in our sample was probably lower than in some other studies. Thirdly, family studies can not provide conclusive evidence for a genetic contribution, as confounding by shared environmental effects (diet and/or microbes being similar in subjects living together) can never be excluded, as suggested by the significant correlation between parent-offspring pairs living together (Table S3 in File S1). Our finding are consistent with the observation that family members tend to have more similar microbiot [42] [43]. Interestingly, the long term diet determines the microbial enterotypes. The *Bacteroides* enterotype was positively associated with animal protein and saturated fats, whereas the *Prevotell*a enterotype was associated with a predominantly plant-based nutrition with high carbohydrates and low meat and dairy consumption [44]. To further study the influence of diet on the generation of indoxyl sulfate and *p*-cresyl sulfate, modulation of the human microbiome enterotype by diet is a logical next step.

The results of the present study must be interpreted within the context of its limitations and strengths. The cross-sectional design precludes conclusions regarding causality of determinants of serum indoxyl sulfate and *p*-cresyl sulfate levels. The ASSOC procedure of SAGE is a validated statistical tool to estimate the heritability of a certain parameter in a population. No conclusions can however be drawn for the individual person within the population. Extrapolation to other, geographically distinct populations also warrants caution. It also should be emphasized that within this statistical context, heritability not exclusively refers to shared genes but also may refer to shared environmental factors. Third, data on dietary intake were unfortunately lacking in the present cohort. As such, we cannot define to what extent diet modifies serum levels of PCS and IndS. Of note, recent data from a cross-sectional study in a cohort of healthy individuals and CKD patients (n = 195), revealed an inverse association between serum indoxyl sulfate levels and dietary fiber intake, independent of renal function (44th American Society of Nephrology Congress, Philadelphia, USA, 10–13/11/2011. Viaene L et al. High dietary fiber intake associates with lower indoxyl sulfate concentrations in chronic kidney disease TH-PO578). We envisage that data from the heritability analysis together with these preliminary dietary data will foster epidemiological studies in nutritionally well-characterized cohorts as well as dietary intervention studies.

In summary, using a targeted approach, we demonstrated that the co-metabolites indoxyl sulfate and *p*-cresyl sulfate exhibit moderate heritability. Besides genetic host factors and environmental factors, also renal function, sex and age influence the serum levels of these co-metabolites. Indoxyl sulfate and *p*-cresyl sulfate may be considered candidate biomarkers of the human microbiome enterotype and may help to explain the link between diet and cardiovascular disease burden. Additional studies are required to confirm these co-metabolites as biomarkers of the human *Bacteroides* enterotype. Whether indoxyl sulfate and *p*-cresyl sulfate can predict cardiovascular risk in the general population above and beyond traditional risk factors also requires further research.

## Supporting Information

File S1 **File S1 contains 3 supplemental tables.**
(DOCX)
