# Supplementary material for: Heritability and Clinical Determinants of Serum Indoxyl Sulfate and p-Cresyl Sulfate, Candidate Biomarkers of the Human Microbiome Enterotype
Source: PLoS One. 2014 May 21;9(5):e79682. doi: 10.1371/journal.pone.0079682 (PMC4029585; doi:10.1371/journal.pone.0079682)
Supplement: File S1 — contains 3 supplemental tables. (DOCX) [file pone.0079682.s001.docx]

Table S1. Indoxyl sulfate and p-cresyl sulfate according to various subgroups

| **Subgroup** (number of subjects) | **Indoxyl sulfate** |  | **p-cresyl sulfate** |
| --- | --- | --- | --- |
| **Sex** |  |  |  |
| Men (384) | 3.20 (1.76–7.60) |  | 12.56 (1.53–41.67) |
| Women (389) | 3.10 (1.72–6.79) |  | 13.31 (1.19–38.77) |
| **Cardiovascular Complications** |  |  |  |
| No (701) | 3.13 (1.75–6.62) |  | 12.89 (1.24–38.32) |
| Yes (72) | 3.43 (1.63–9.60)° |  | 14.25 (2.13–56.20) |
| **Smoking** |  |  |  |
| No (616) | 3.25 (1.73–7.40) |  | 13.90 (1.20–41.77) |
| Yes (157) | 2.75 (1.71–6.61)‡ |  | 10.54 (2.13–27.86)† |
| **Alcohol Intake** |  |  |  |
| No (240) | 3.26 (1.80–8.63) |  | 14.83 (1.34–53.17) |
| Yes (533) | 3.05 (1.70–6.54)* |  | 12.46 (1.46–37.74)* |
| **Antihypertensive Drug Intake** |  |  |  |
| No (578) | 3.03 (1.72–6.41) |  | 12.18 (1.29–34.32) |
| Yes (195) | 3.44 (1.77–9.36)‡ |  | 16.23 (2.13–53.62)‡ |
| **Diabetic** |  |  |  |
| No (746) | 3.13 (1.73–7.10) |  | 12.89 (1.46–38.94) |
| Yes (27) | 3.96 (1.98–9.36)* |  | 16.35 (1.02–105.84) |

Values are medians (5^th^ to 95^th^ percentile interval).

Significance of between-group differences according to the 2-sample Wilcoxon test: °*P*=0·054; * *P<*0·05; † *P<*0·01; and ‡ *P<*0·001.

Table S2. Pearson Correlation Coefficients between Indoxyl sulfate and p-cresyl sulfate and various covariates

| **Subgroup** (number of subjects) | **Log Indoxyl Sulfate** |  | **Log p-Cresyl Sulfate** |
| --- | --- | --- | --- |
| Age | 0.173‡ |  | 0.194‡ |
| Systolic Blood Pressure | 0.152‡ |  | 0.053 |
| Diastolic Blood Pressure | 0.052 |  | -0.018 |
| Body Mass Index | 0.090* |  | 0.022 |
| Total Cholesterol | 0.069° |  | 0.070° |
| Log Triglycerides | 0.193‡ |  | 0.041 |
| Serum Creatinine | -0.014 |  | -0.079* |
| Measured Creatinine Clearance | -0.108† |  | -0.122‡ |
| Calculated Creatinine Clearance | -0.169‡ |  | -0.188‡ |

Values are Pearson Correlation Coefficients.

Significance of the Correlation Coefficients: ° *P<*0·06; * *P<*0·05; † *P<*0·01; and ‡ *P<*0·001.

# Table S3. Proportion of variance in p-cresyl sulphate and indoxyl sulphate explained by polygenic and shared environmental effects.

|  | Heritability | *P* |  | Shared Environment | *P* |
| --- | --- | --- | --- | --- | --- |
| Indoxyl sulfate | 0.19 | 0.023 |  | 0.11 | 0.10 |
| P-cresyl sulfate | 0.22 | 0.023 |  | 0.17 | 0.049 |

Values are proportions. Shared environment is estimated from the effect shared by spouses.

Estimates are adjusted for age, triglycerides, current smoking status, creatinine clearance and history of cardiovascular complications.
